# Impact of probiotic intake on cortisol concentration as a physiological marker of mental health: Meta-analysis.

**DOI:** 10.1192/j.eurpsy.2026.11240

**Published:** 2026-07-31

**Authors:** A. Chouchane, S. Ben Fredj, H. Kechiche, R. Ghammem, O. ElMaalel, A. Gaddour, A. Skhiri, N. Zammit, M. Makhloufi, M. Bouhoula, A. Aloui, I. Kacem, A. Brahem, H. Kalboussi, S. Chatti, I. Harrabi

**Affiliations:** 1 Occupational Medicine and Professional Pathologies Department, Farhat Hached Teaching Hospital; 2 Faculty of Medicine of Sousse, Research Laboratory LR19SP03, University of Sousse; 3 Department of Epidemilogy, Farhat Hached Teaching Hospital, Sousse; 4 Occupational Medicine and Professional Pathologies Department, Ibn El Jazzar teaching Hospital, Kairouan, Tunisia

## Abstract

**Introduction:**

Chronic stress is a major risk factor for physical and mental health problems. Cortisol is widely recognized as a key physiological marker of stress. Probiotics, through their action on the gut–brain axis, have been suggested as a promising approach to support stress regulation.

**Objectives:**

To examine the impact of probiotic supplementation on cortisol levels, as a physiological marker of stress, in healthy working adults.

**Methods:**

We conducted a comprehensive systematic search of multiple databases, including Embase, The Cochrane Library, and PubMed via Medline, covering the period from December 2023 to April 2024. The search terms were developed from previous reviews and refined in collaboration with a specialist librarian using the PRESS (Peer Review of Electronic Search Strategies) checklist to ensure accuracy and completeness. The six PICOst dimensions (participants, interventions, comparators, outcomes, study design, and timing) were applied to define the eligibility criteria. We exclusively considered randomized controlled trials. Only studies examining the effect of probiotic supplementation on cortisol levels were included.

**Results:**

A total of five trials were identified as meeting the inclusion criteria, involving 437 participants. Occupational groups varied across the records included, with the majority of randomised workers being health professionals. The standardized mean difference of -0.26 (95% CI [-0.45, -0.08]) provided by the meta-analysis suggested a statistically significant decrease in cortisol levels among participants who received probiotic supplementation compared to those in the control groups. Furthermore, the heterogeneity analysis yielded an I² value of 0%, indicating a lack of significant variability in the results across the included studies. This homogeneity enhances the reliability of the combined effect size, suggesting that the observed reduction in cortisol levels is likely a reproducible outcome of probiotic supplementation rather than a consequence of variations in study populations, interventions, or methodological approaches.

**Conclusions:**

These findings indicate that probiotic supplementation may effectively reduce cortisol levels, a key physiological marker of stress, in healthy adults. The consistency across studies strengthens the evidence for a potential role of probiotics in stress modulation.

**Disclosure of Interest:**

None Declared

